# *PSCA* s2294008 C>T and rs2976392 G>A polymorphisms contribute to cancer susceptibility: evidence from published studies

**DOI:** 10.18632/genesandcancer.63

**Published:** 2015-05

**Authors:** Yong Gu, Qiang-Sheng Dai, Rui-Xi Hua, Bing Zhang, Jin-Hong Zhu, Jian-Wen Huang, Bin-Hui Xie, Shi-Qiu Xiong, Guo-Sheng Tan, He-Ping Li

**Affiliations:** ^1^ Department of Thoracic Surgery, The First Affiliated Hospital of Sun Yat-sen University, Guangzhou, Guangdong, China; ^2^ Department of Oncology, The First Affiliated Hospital of Sun Yat-sen University, Guangzhou, Guangdong, China; ^3^ Department of Medical Imaging, The First Affiliated Hospital of Sun Yat-sen University, Guangzhou, Guangdong, China; ^4^ Molecular Epidemiology Laboratory and Laboratory Medicine, Harbin Medical University Cancer Hospital, Harbin, Heilongjiang, China; ^5^ Department of Radiotherapy, The First Affiliated Hospital of Sun Yat-sen University, Guangzhou, Guangdong, China; ^6^ Department of Hepatobiliary Surgery, The First Affiliated Hospital of Gannan Medical University, Ganzhou, Jiangxi, China; ^7^ Department of Biochemistry, University of Leicester, Leicester, UK

**Keywords:** PSCA, GWAS, polymorphism, susceptibility, meta-analysis

## Abstract

*PSCA* gene plays an important role in cell adhesion, proliferation and survival. Increasing studies have focused on the association of *PSCA* gene rs2294008 C>T and rs2976392 G>A with cancer risk. However, the conclusions were inconsistent. Therefore, we performed a meta-analysis to elucidate whether there is a true association, or artifact. We systematically searched eligible studies from MEDLINE, EMBASE and CBM database. Odds ratios and 95% confidence intervals were used to evaluate the strength of the association. The final analysis included 32 studies consisting of 30028 cases and 38765 controls for the rs2294008 C>T polymorphism, and 14 studies with 8190 cases and 7176 controls for the rs2976392 G>A polymorphism. Consequently, the *PSCA* rs2294008 C>T polymorphism was significantly associated with increased overall cancer risk. Further stratifications indicated the increased risk was more pronounced for gastric (diffused type and non-gastric cardia adenocarcinoma) and bladder cancer. A similar association was observed for the rs2976392 G>A polymorphism. This meta-analysis demonstrated that both of the *PSCA* rs2294008 C>T and rs2976392 G>A polymorphisms are associated with increased cancer risk, especially for gastric cancer and bladder cancer. Further large-scale studies with different ethnicities and subtypes of gastric cancer are required to confirm the results from this meta-analysis.

## INTRODUCTION

Despite the significant improvements in the early detection and treatment, cancer still remains a major public health burden worldwide, with approximately 12.7 million new cases and 7.6 million new deaths in 2008 [1]. Cancer is a multi-step complex disease involving both genetic and environmental factors [2]. Extensive evidence has indicated the important roles of polymorphisms in the key genes during the process of carcinogenesis [3-5]. Screening and identification of single nucleotide polymorphisms (SNPs) which are related to cancer susceptibility would greatly benefit individuals at high risk for cancer in the early prevention and treatment settings. Genome-wide association studies (GWASs) which interrogate a large number of tagging SNPs in a high density across the whole genome, have provided a robust tool to discover novel cancer susceptibility loci or genes [6].

Over the past decade, GWAS has successfully identified hundreds of genetic markers that are related to the susceptibility to a wide spectrum of diseases including cancers [7]. In a two-stage GWAS on gastric cancer conducted in Japanese and Korean population, two SNPs (rs2294008 C>T and rs2976392 G>A) in the *prostate stem cell antigen* (*PSCA*) gene were found to be significantly associated with increased stomach cancer risk [8]. Wu et al. [9] also found the *PSCA* rs2294008 C>T polymorphism was a bladder cancer susceptibility locus in a three-stage GWAS.

The *PSCA* gene is located on chromosome 8q24.2, encoding a 123-amino acid glycoprotein, a cell surface antigen. PSCA was first identified as a prostate-specific antigen that is over-expressed in prostate cancer [10], and plays an important role in cell adhesion, proliferation, and survival [11]. It is also expressed in other solid tumors, including ovarian mucinous, pancreatic cancer, renal-cell carcinoma and bladder cancer [10, 12]. In contrast with observations in prostate cancer, PSCA expression is down-regulated in several cancers including bladder cancer and gastric cancer [13].

The associations of the *PSCA* rs2294008 C>T and rs2976392 G>A polymorphisms and cancer susceptibility have been widely investigated. The significant associations were reported and validated in some studies among different ethnic populations and different types of cancers [8, 9, 14-43]; however, others failed to replicate such association. The controversy is probably due to different ethnicities, histology types, and relatively small sample size in individual studies. The previous meta-analysis mainly focus on gastric cancer [44-49] and bladder cancer [50]. Until now, few meta-analysis has been performed to investigate the association of *PSCA* rs2294008 C>T and rs2976392 G>A polymorphisms with overall cancer risk. With this in mind we conducted the present meta-analysis to clarify the role of these two polymorphisms in carcinogenesis.

## RESULTS

### Study characteristics

**Figure 1:**
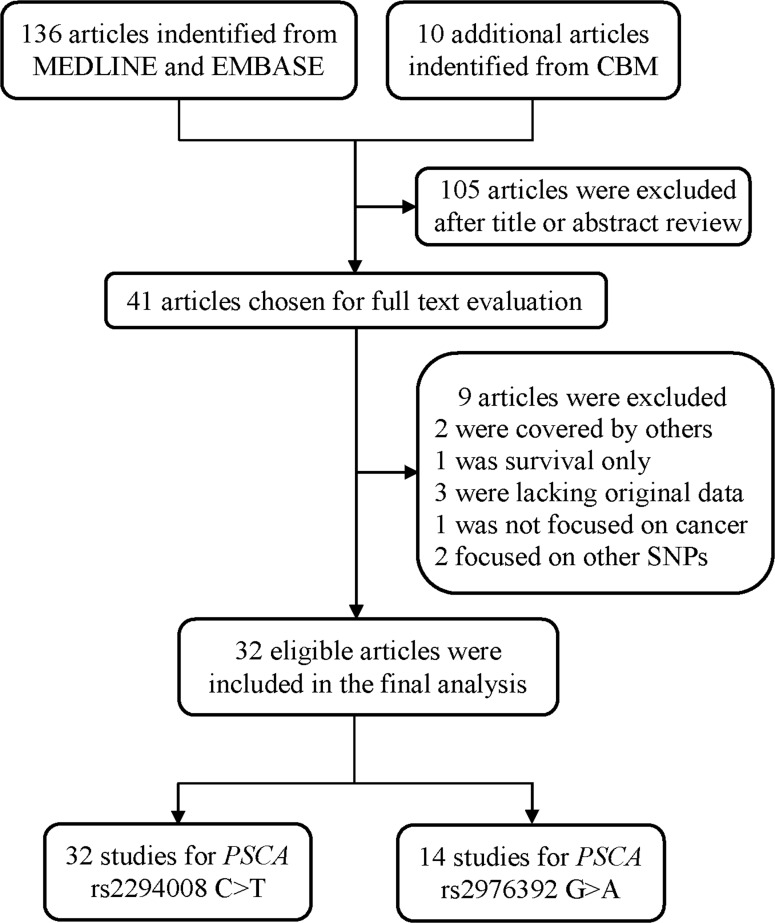
Flow diagram of included studies for the association between PSCA polymorphisms and overall cancer susceptibility

As shown in Figure 1, a total of 136 publications were indentified from PubMed and Embase. Moreover, 10 additional publications were indentified from CBM database. After reviewing the titles and abstracts of the potential available articles, 105 publications were excluded, mainly due to no relevance, reviews, or functional studies. Forty one full-text articles that met the crude inclusion criteria were further evaluated for eligibility. Of them, two studies written in Chinese were excluded because of the overlap of study subjects [22, 32]. Moreover, one investigation was excluded for only focused on gastric survival not case-control study [51], three publications were excluded due to lack of genotyping data [52-54], one was not focused on cancer [55], and another two that did not pertain to either of these two polymorphism were also excluded [56, 57]. Eventually, 32 publications were included in the final meta-analysis.

**Figure 2:**
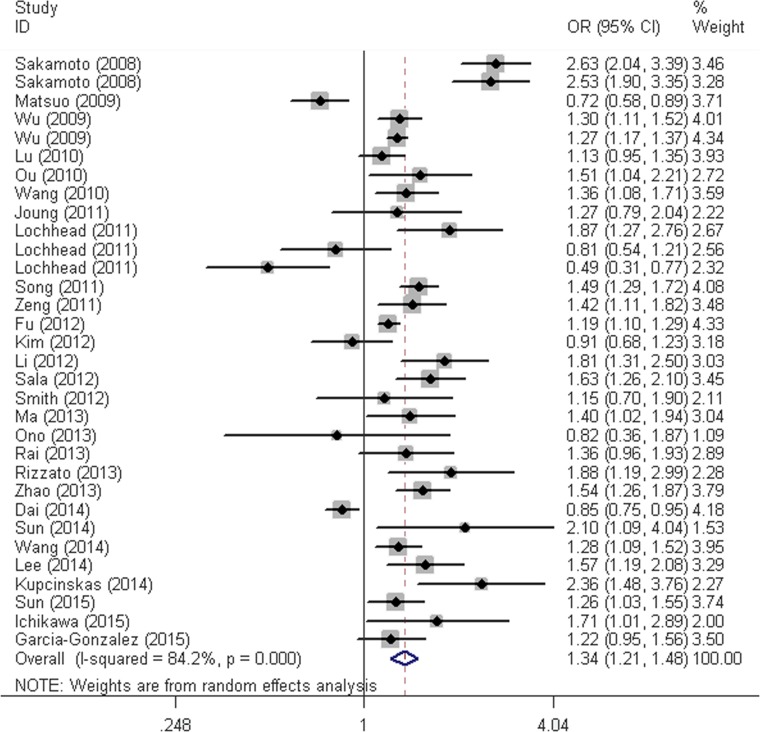
Forest plot for overall cancer risk associated with the PSCA rs2294008 C>T polymorphism by dominant model (CT/TT vs. CC) For each study, the estimation of OR and its 95% CI are plotted with a box and a horizontal line. ◇, pooled ORs and its 95% CIs.

Investigations with multiple source of subjects and multiple types of cancers [8, 20] were considered as multiple studies. Overall, there were 32 studies investigating the association between *PSCA* rs2294008 C>T polymorphism and cancer susceptibility, consisting of 30028 cases and 38765 controls [8, 9, 14-35, 39-43], and 14 studies performed on the relation between *PSCA* rs2976392 G>A polymorphism and overall cancer susceptibility, with 8190 cases and 7176 controls [8, 14-17, 19, 24, 29, 36-39, 43]. The main characteristics of the studies included in this meta-analysis were summarized in Table 1.

**Table 1:** Characteristics of studies included in the current meta-analysis

Surname	Year	Cancer	Country	Ethnicity	Source	Genotype method	Case				Control				MAF	HWE
rs2294008 C>T polymorphism							CC	CT	TT	all	CC	CT	TT	all		
Sakamoto	2008	gastric	Japan	Asian	HB	GWAS	96	700	728	1524	210	650	536	1396	0.62	0.574
Sakamoto	2008	gastric	Korea	Asian	HB	Taqman	133	461	277	871	122	176	92	390	0.46	0.069
Matsuo	2009	gastric	Japan	Asian	HB	Taqman	330	329	49	708	273	338	97	708	0.38	0.638
Wu	2009	gastric	China	Asian	PB	PCR-RFLP	759	819	132	1710	506	412	77	995	0.28	0.587
Wu	2009	bladder	USA&European	Caucasian	HB	GWAS	1288	2613	1137	5038	2842	4668	1853	9363	0.45	0.418
Lu	2010	gastric	China	Asian	PB	PCR-RFLP	547	404	72	1023	605	387	77	1069	0.25	0.166
Ou	2010	gastric	China	Asian	HB	PCR/LDR	85	93	18	196	132	96	18	246	0.27	0.924
Wang	2010	bladder	China	Asian	HB	PCR-RFLP	272	259	50	581	316	220	44	580	0.27	0.508
Joung	2011	prostate	Korea	Asian	HB	MassARRAY	45	98	49	192	47	84	37	168	0.47	0.963
Lochhead	2011	gastric	Poland	Caucasian	PB	Taqman	47	143	102	292	101	166	115	382	0.52	0.011
Lochhead	2011	gastric	USA	Caucasian	PB	Taqman	85	129	94	308	49	110	49	208	0.50	0.405
Lochhead	2011	esophageal	USA	Caucasian	PB	Taqman	61	63	34	158	49	110	49	208	0.50	0.405
Song	2011	gastric	Korea	Asian	HB	PCR-RFLP	576	1620	1049	3245	414	818	468	1700	0.52	0.130
Zeng	2011	gastric	China	Asian	HB	PCR-RFLP	202	216	42	460	289	223	37	549	0.27	0.493
Fu	2012	bladder	European&USA	Caucasian	PB	GWAS	1363	2804	1226	5393	2107	3645	1572	7324	0.46	0.952
Kim	2012	breast	Korea	Asian	HB	MassARRAY	119	216	116	451	113	240	106	459	0.49	0.324
Li	2012	gastric	China	Asian	PB	MassARRAY	124	141	35	300	168	111	21	300	0.26	0.650
Sala	2012	gastric	European	Caucasian	PB	Taqman	93	198	118	409	491	714	310	1515	0.44	0.088
Smith	2012	colorectal	UK	Caucasian	HB	Taqman	25	39	13	77	287	387	130	804	0.40	0.130
Ma	2013	bladder	China	Asian	PB	MassARRAY	84	80	11	175	543	355	64	962	0.25	0.563
Ono	2013	gallbladder	Japan	Asian	HB	Taqman	9	23	12	44	30	75	68	173	0.61	0.242
Rai	2013	gallbladder	India	Asian	HB	Taqman	104	233	68	405	79	126	42	247	0.43	0.493
Rizzato	2013	gastric	Germany	Caucasian	PB	Taqman	23	86	69	178	231	507	319	1057	0.54	0.269
Zhao	2013	gastric	China	Asian	PB	DHPLC	275	342	100	717	465	401	85	951	0.30	0.913
Dai	2014	esophageal	China	Asian	PB	Taqman	1232	724	127	2083	1222	851	147	2220	0.26	0.944
Sun	2014	gastric	USA	African	HB	Taqman	17	64	49	130	30	63	32	125	0.51	0.926
Wang	2014	bladder	China	Asian	PB	Taqman	604	509	97	1210	566	376	66	1008	0.25	0.739
Lee	2014	bladder	Korea	Asian	HB	HRM	70	222	119	411	414	818	468	1700	0.52	0.130
Kupcinskas	2014	gastric	Lithuania	Caucasian	HB	Taqman	33	116	102	251	64	123	56	243	0.48	0.834
Sun	2015	gastric	China	Asian	HB	Taqman	322	309	61	692	405	297	72	774	0.28	0.105
Ichikawa	2015	gastric	Japan	Asian	HB	PCR-RFLP	24	104	65	193	52	119	95	266	0.58	0.185
Garcia-Gonzalez	2015	gastric	Spain	Caucasian	HB	Taqman	154	302	147	603	199	346	130	675	0.45	0.349
rs2976392 G>A polymorphism							GG	AG	AA	all	GG	AG	AA	all		
Sakamoto	2008	gastric	Japan	Asian	HB	GWAS	97	691	737	1525	211	650	536	1397	0.62	0.545
Sakamoto	2008	gastric	Korea	Asian	HB	Taqman	134	453	278	865	122	175	93	390	0.46	0.054
Matsuo	2009	gastric	Japan	Asian	HB	Taqman	331	328	48	707	274	337	96	707	0.37	0.635
Wu	2009	gastric	China	Asian	PB	PCR-RFLP	789	793	142	1724	492	429	81	1002	0.29	0.350
Lu	2010	gastric	China	Asian	PB	PCR-RFLP	500	464	79	1043	602	402	78	1082	0.26	0.336
Ou	2010	gastric	China	Asian	HB	PCR/LDR	99	85	12	196	130	102	14	246	0.26	0.298
Joung	2011	prostate	Korea	Asian	HB	MassARRAY	45	100	49	194	46	85	37	168	0.47	0.848
Shen	2011	gastric	China	Asian	PB	DHPLC	24	31	5	60	29	26	5	60	0.30	0.806
Kim	2012	breast	Korea	Asian	HB	MassARRAY	121	217	115	453	115	239	106	460	0.49	0.397
Ono	2013	gallbladder	Japan	Asian	HB	Taqman	9	23	12	44	29	76	68	173	0.61	0.328
Ju	2013	gastric	China	Asian	HB	sequencing	67	65	23	155	107	87	16	210	0.28	0.771
Wang	2014	gastric	China	Asian	HB	Taqman	131	134	18	283	149	108	18	275	0.26	0.791
Kupcinskas	2014	gastric	Lithuania	Caucasian	HB	Taqman	34	113	102	249	62	116	54	232	0.48	0.986
Sun	2015	gastric	China	Asian	HB	Taqman	319	308	65	692	403	299	72	774	0.29	0.130

HB, Hospital based; PB, Population based; GWAS, Genome wide association study; PCR-RFLP, Polymorphism chain reaction-restriction fragment length polymorphism; PCR-LDR, Polymorphism chain reaction-ligase detection reaction; DHPLC, Denaturing high performance liquid chromatography; HRM, High resolution melting; MAF, Minor allele frequency; HWE, Hardy-Weinberg equilibrium.

For the *PSCA* rs2294008 C>T polymorphism, the number of cases varied from 44 to 5393 while the range of controls fell between 125 and 9363 among studies. The studied cancer type included gastric cancer [8, 14-17, 20-22, 25, 26, 31, 32, 34, 39, 41-43], bladder cancer [9, 18, 23, 28, 35, 40], prostate cancer [19], esophageal cancer [20, 33], breast cancer [24], colorectal cancer [27], and gallbladder cancer [29, 30]. In term of ethnicity, 21 studies were performed among Asians, 10 studies among Caucasians, and one study among Africans. Of these studies, 19 were hospital-based, 13 were population-based. Intriguingly, in the studies focused on gastric cancer, 13 studies provided detailed genotype frequency data by the gastric cancer subtypes, and six studies by sites. For the *PSCA* rs2976392 G>A polymorphism, the number of cases ranged from 44 to 1525 while varying control sample sizes (from 60 to 1397) were also observed among selected studies. The studied cancer type included gastric cancer [8, 14-17, 36-39, 43], prostate cancer [19], breast cancer [24], and gallbladder cancer [29]. Of them, 13 studies were performed among Asians and one among Caucasians. Eleven of them were hospital-based, while three were population-based.

### Meta-analysis results

Results of the association between these two polymorphisms and cancer risk were summarized in Table 2. We found that the *PSCA* rs2294008 C>T polymorphism was associated with an increased overall cancer risk (homozygous: OR = 1.41, 95% CI = 1.23-1.61; heterozygous: OR = 1.31, 95% CI = 1.19-1.43, recessive: OR = 1.19, 95% CI = 1.10-1.29, dominant: OR = 1.34, 95% CI = 1.21-1.48, and allele comparison: OR = 1.20, 95% CI = 1.13-1.28). The stratification analyses by cancer types found that carriers of *PSCA* rs2294008 T had a significantly increased risk of gastric cancer (homozygous: OR = 1.64, 95% CI = 1.32-2.03; heterozygous: OR = 1.44, 95% CI = 1.26-1.64, recessive: OR = 1.28, 95% CI = 1.12-1.46, dominant: OR = 1.51, 95% CI = 1.30-1.75, and allele comparison: OR = 1.29, 95% CI = 1.18-1.41) and increased risk of bladder cancer (homozygous: OR = 1.29, 95% CI = 1.21-1.38; heterozygous: OR = 1.25, 95% CI = 1.18-1.32, recessive: OR = 1.13, 95% CI = 1.07-1.19, dominant: OR = 1.26, 95% CI = 1.20-1.32, and allele comparison: OR = 1.14, 95% CI = 1.11-1.18). Stratification analyses also elucidated that this polymorphism increased cancer risk among Asians, Caucasians as well as Africans by ethnicity. Moreover, increased cancer risk associated with the SNP was also observed in population-based and hospital-based studies by the source of controls. When studies were stratified by subtypes of gastric cancer, we found the increased risk was more pronounced for diffuse type (homozygous: OR = 2.45, 95% CI = 1.68-3.57; heterozygous: OR = 1.72, 95% CI = 1.28-2.30, recessive: OR = 1.67, 95% CI = 1.37-2.03, dominant: OR = 1.93, 95% CI = 1.41-2.63, and allele comparison: OR = 1.52, 95% CI = 1.31-1.78) than intestinal type (homozygous: OR = 1.42, 95% CI = 1.05-1.92; heterozygous: OR = 1.36, 95% CI = 1.13-1.62, recessive: OR = 1.19, 95% CI = 0.98-1.44, dominant: OR = 1.39, 95% CI = 1.13-1.71, and allele comparison: OR = 1.22, 95% CI = 1.06-1.41). We also observed that *PSCA* rs2294008 polymorphism conferred higher susceptibility to NGCA than the GCA subtype.

**Table 2:** Meta-analysis of the association between PSCA rs2294008 C>T and rs2976392 G>A polymorphisms and cancer risk

Variables	No. of studies	Homozygous		Heterozygous		Recessive		Dominant		Allele Comparing	
		OR (95% CI)	*P* het	OR (95% CI)	*P* het	OR (95% CI)	*P* het	OR (95% CI)	*P* het	OR (95% CI)	*P* het
rs2294008 C>T	TT vs. CC		CT vs. CC		TT vs. (CT + CC)		(CT + TT) vs. CC		T vs. C	
All	32	1.41 (1.23-1.61)	<0.001	1.31 (1.19-1.43)	<0.001	1.19 (1.10-1.29)	<0.001	1.34 (1.21-1.48)	<0.001	1.20 (1.13-1.28)	<0.001
Cancer type											
Gastric	19	1.64 (1.32-2.03)	<0.001	1.44 (1.26-1.64)	<0.001	1.28 (1.12-1.46)	<0.001	1.51 (1.30-1.75)	<0.001	1.29 (1.18-1.41)	<0.001
Bladder	6	1.29 (1.21-1.38)	0.572	1.25 (1.18-1.32)	0.356	1.13 (1.07-1.19)	0.676	1.26 (1.20-1.32)	0.414	1.14 (1.11-1.18)	0.423
Others	7	0.95 (0.77-1.17)	0.238	0.93 (0.74-1.18)	0.012	0.99 (0.85-1.15)	0.634	0.94 (0.75-1.17)	0.012	0.96 (0.85-1.09)	0.054
Ethnicity											
Asian	21	1.34 (1.09-1.64)	<0.001	1.35 (1.19-1.54)	<0.001	1.11 (0.98-1.25)	<0.001	1.36 (1.18-1.56)	<0.001	1.18 (1.08-1.29)	<0.001
Caucasian	10	1.48 (1.23-1.78)	<0.001	1.20 (1.03-1.39)	<0.001	1.30 (1.15-1.47)	0.002	1.28 (1.10-1.49)	<0.001	1.21 (1.11-1.34)	<0.001
African	1	2.70 (1.29-5.68)	/	1.79 (0.90-3.57)	/	1.76 (1.03-3.01)	/	2.10 (1.09-4.04)	/	1.60 (1.13-2.28)	/
Source of control											
HB	19	1.46 (1.20-1.78)	<0.001	1.39 (1.22-1.57)	<0.001	1.18 (1.04-1.33)	<0.001	1.42 (1.23-1.64)	<0.001	1.21 (1.11-1.32)	<0.001
PB	13	1.33 (1.10-1.60)	<0.001	1.21 (1.05-1.40)	<0.001	1.20 (1.07-1.36)	0.010	1.25 (1.08-1.44)	<0.001	1.18 (1.07-1.30)	<0.001
Subtype											
Intestinal	13	1.42 (1.05-1.92)	<0.001	1.36 (1.13-1.62)	<0.001	1.19 (0.98-1.44)	<0.001	1.39 (1.13-1.71)	<0.001	1.22 (1.06-1.41)	<0.001
Diffuse	13	2.45 (1.68-3.57)	<0.001	1.72 (1.28-2.30)	<0.001	1.67 (1.37-2.03)	0.002	1.93 (1.41-2.63)	<0.001	1.52 (1.31-1.78)	<0.001
Sites											
GCA	6	1.14 (0.90-1.43)	0.660	1.18 (1.02-1.36)	0.712	1.05 (0.85-1.30)	0.546	1.17 (1.02-1.34)	0.765	1.10 (1.00-1.22)	0.822
NGCA	6	1.54 (1.31-1.81)	0.143	1.39 (1.25-1.55)	0.743	1.32 (1.15-1.52)	0.185	1.42 (1.28-1.58)	0.583	1.28 (1.19-1.38)	0.327
rs2976392 G>A	AA vs. GG	AG vs. GG	AA vs. (AG + GG)	(AG + AA) vs. GG	A vs. G
All	14	1.35 (0.95-1.91)	<0.001	1.32 (1.10-1.58)	<0.001	1.14 (0.92-1.41)	<0.001	1.35 (1.09-1.66)	<0.001	1.19 (1.03-1.37)	<0.001
Cancer type											
Gastric	11	1.47 (0.97-2.22)	<0.001	1.40 (1.14-1.71)	<0.001	1.18 (0.91-1.52)	<0.001	1.44 (1.13-1.83)	<0.001	1.24 (1.06-1.46)	<0.001
Others	3	1.04 (0.75-1.45)	0.323	0.95 (0.74-1.23)	0.547	1.03 (0.74-1.44)	0.215	0.98 (0.77-1.24)	0.477	1.01 (0.83-1.22)	0.251
Ethnicity											
Asian	13	1.25 (0.88-1.79)	<0.001	1.29 (1.07-1.56)	<0.001	1.07 (0.87-1.33)	<0.001	1.30 (1.05-1.61)	<0.001	1.15 (1.00-1.32)	<0.001
Caucasian	1	3.44 (2.02-5.87)	/	1.78 (1.09-2.91)	/	2.29 (1.54-3.40)	/	2.31 (1.45-3.67)	/	1.88 (1.45-2.43)	/
Source of control											
HB	11	1.40 (0.89-2.18)	<0.001	1.32 (1.03-1.70)	<0.001	1.16 (0.89-1.51)	<0.001	1.36 (1.01-1.83)	<0.001	1.19 (0.99-1.44)	<0.001
PB	3	1.15 (0.92-1.43)	0.889	1.26 (1.10-1.45)	0.297	1.03 (0.84-1.28)	0.988	1.25 (1.09-1.42)	0.314	1.15 (1.05-1.25)	0.426
Subtype											
Intestinal	4	1.85 (1.16-2.93)	0.004	1.55 (1.26-1.90)	0.209	1.31 (1.00-1.73)	0.039	1.67 (1.26-2.22)	0.034	1.36 (1.15-1.61)	0.025
Diffuse	4	3.30 (2.11-5.14)	0.022	2.40 (1.43-4.03)	<0.001	1.66 (1.45-1.91)	0.984	2.64 (1.51-4.59)	<0.001	1.67 (1.48-1.89)	0.231

HB, hospital based; PB, population based; GCA, gastric cardia adenocarcinoma; NGCA, non-gastric cardia adenocarcinoma.

Similarly, we also found that *PSCA* rs2976392 G>A polymorphism was associated with increased overall cancer risk (heterozygous model: OR = 1.32, 95% CI = 1.10-1.58, dominant model: OR = 1.35, 95% CI = 1.09-1.66, and allele comparing: OR = 1.19, 95% CI = 1.03-1.37). In the stratification analyses, the significant association was observed for gastric cancer (heterozygous model: OR = 1.40, 95% CI = 1.14-1.71, dominant model: OR = 1.44, 95% CI = 1.13-1.83, and allele comparing: OR = 1.24, 95% CI = 1.06-1.46), Asians, hospital-based studies, population-based studies as well as both the diffuse and intestinal types. Interestingly, the risk effect of the SNP on the diffuse type was more evident than the intestinal type.

### Heterogeneity and sensitivity analyses

As shown in Table 2, we observed that there existed statistically significant between-study heterogeneity for both of these two polymorphisms, thus the random-effect model was chosen for all the analysis to generated wider CIs for all genetics models. We subsequently conducted sensitivity analysis to investigate the influence of individual study on the pooled risk estimates by omitting studies from the pooled analysis individually. Omitting each of studies did not qualitatively influence the corresponding pooled ORs in overall analysis and subgroups, suggesting that the results were statistically robust (data not shown).

### Publication bias

The Begg's funnel test and Egger's linear regression test were performed to quantitatively evaluate the publication bias of the meta-analysis. The shape of the funnel plots did not reveal any evidence of obvious asymmetry, and the Egger's test for *PSCA* rs2294008 C>T indicated that there was no publication bias in the current meta-analysis (homozygous: *P* = 0.616, heterozygous: *P* = 0.209, recessive: *P* = 0.930, dominant: *P* = 0.186, and allele comparison: *P* = 0.385). Likely, no publication bias was found for the *PSCA* rs2976392 G>A polymorphism (homozygous: *P* = 0.564, heterozygous: *P* = 0.733, recessive: *P* = 0.263, dominant: *P* = 0.623, and allele comparison: *P* = 0.747).

## DISCUSSION

In the current updated meta-analysis, we comprehensively evaluated the association of *PSCA* rs2294008 C>T and rs2976392 G>A polymorphisms with overall cancer susceptibility by pooling together 32 studies with 30028 cases and 38765 controls for the rs2294008 C>T polymorphism, and 14 studies with 8190 cases and 7176 controls for the rs2976392 G>A polymorphism. We found both of the *PSCA* rs2294008T and rs2976392A carriers were associated with increased risk of overall cancer, especially the former. We also confirmed that the associations were more obvious for gastric cancer and bladder cancer, especially diffuse type and NGCA for gastric cancer.

**Figure 3:**
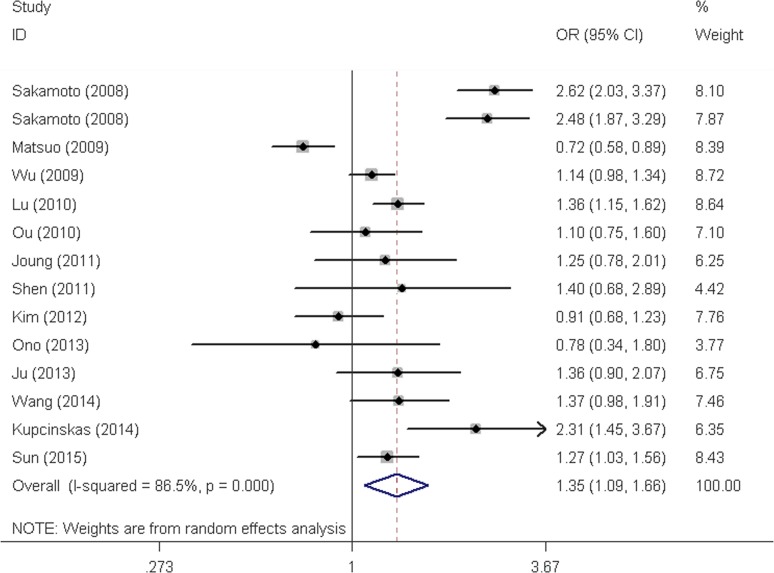
Forest plot for overall cancer risk associated with the PSCA rs2976392 G>A polymorphism by dominant model (AG/AA vs. GG) For each study, the estimation of OR and its 95% CI are plotted with a box and a horizontal line.◇, pooled ORs and its 95% CIs.

The *PSCA* gene, located on chromosome 8q24.2, encodes a PSCA protein that is a cell surface antigen. PSCA belongs to the LY-6/Thy-1 family and is highly expressed in normal prostate and further up-regulated in prostate cancer [10], which is also found in non-prostatic malignancies including gastric cancer [8]. This protein plays a critical role in multiple important cellular events, such as adhesion, proliferation, and survival [11]. In 2010, a two-stage gastric cancer GWAS conducted among Japanese and Korean subjects demonstrated that the rs2976392 G>A and rs2294008 C>T polymorphisms in the *PSCA* gene significantly increased stomach cancer risk [8]. Further *in vitro* experiments revealed that the *PSCA* rs2294008T variant might decrease the transcriptional activity transcription of the host gene by modulating its upstream fragment [8]. Moreover, the *PSCA* gene rs2294008 C>T polymorphism was also found to be associated with increased bladder cancer risk by a three-stage GWAS with a total of 5038 cases and 9363 controls [9].

The association between *PSCA* polymorphisms and cancer risk discovered by previous GWASs were extensively validated among different ethnic populations and different types of cancers. For example, Wu et al. [15] reported that the association of the *PSCA* rs2294008 C>T polymorphism and gastric cancer risk was more prominent among patients with non-cardia gastric cancer than cardia gastric cancer with a total of 1736 cases and 1020 controls. The significant association was also validated by studies conducted among different ethnicities worldwide [14, 17, 21, 22, 25, 26, 52-55]. However, some studies failed to replicate the association [16, 20]. To resolve the controversy, six meta-analyses have been performed to evaluate the relationship between *PSCA* polymorphisms and gastric cancer susceptibility [44-49], to date. Qiao et al. [44] included eight studies from seven publications with a total of 9738 gastric cancer cases and 7054 controls, and concluded that rs2294008 T allele and rs2976392 A allele were significantly associated with increased gastric cancer risk. These findings were confirmed by other meta-analysis [45-48]. The most recent meta-analysis by Gu et al. [49] involved 16 studies. They found that the *PSCA* rs2294008T carriers had a 1.46-fold increased gastric cancer risk when compared to the rs2294008C carriers. They also found the rs2976392A carriers had a 1.49-fold increased cancer risk when compared to non-carriers. Only one of the previous meta-analyses explored the association between polymorphisms of *PSCA* gene and bladder cancer [50], which included a total of four studies with 9617 cases and 16323 controls. They found that the rs2294008 C>T polymorphism was associated with increased bladder cancer under all the genetic models.

In the current meta-analysis, we included all the studies investigating the association between *PSCA* polymorphisms and cancer risk, i.e., a total of 32 studies with 30028 cases and 38765 controls for the *PSCA* rs2294008 C>T polymorphism, and 14 studies with 8190 cases and 7176 controls for the *PSCA* rs2976392 G>A polymorphism. To the best of our knowledge, this is the largest and most comprehensive meta-analysis for the association of interest. We found the *PSCA* rs2294008 C>T polymorphism was associated with overall cancer risk, and the association remained significant among all studies ethnicities and subgroups by source of controls. We also confirmed that this risk was more predominant for bladder cancer and gastric cancer, especially the diffuse gastric cancer and non-gastric cardia adenocarcinoma. The *PSCA* rs2976392 G>A polymorphism was found to associate with overall cancer risk mainly under heterozygous model and dominant model. The association was also valid for gastric cancer, Asians and population-based studies, and was more evident for diffuse subtype. The *PSCA* rs2294008 C>T polymorphism was associated with gastric cancer and bladder cancer, which may be ascribed to cancer specificity. Intestinal type may be caused by excessive salt intake, alcohol consumption, and deficiency of fresh fruit and vegetable, while the diffuse type may be associated with gastroesophageal reflux disease and obesity [58]. Gastric cardia cancer arises from the gastroesophageal junction and may differ from non-gastric cardia cancer regarding epidemiological characteristics and clinical features [53]. Susceptibility to different subtypes of cancer may vary [59, 60]. Therefore, the association with diffuse subtype and non-gastric cardia cancer appeared to be biology plausible. Moreover, the association with the *PSCA* rs2976392 G>A polymorphism was only found for gastric cancer, Asians and population-based controls, which might be ascribed to the limited number of investigations, since all the 12 studies were performed among Asian and nine of them investigated gastric cancer. So the findings for this polymorphism should be interpreted cautiously.

Several limitations should be stated in this meta-analysis. First, substantial between-study heterogeneity, not explained by sensitivity analysis, was observed in the current meta-analysis, which might due to limited study number. Thus, we chose random-effects model for all genetic models to produce wider CIs. Second, although we had included 32 publications, the sample size remained relatively small for certain cancer types and ethnicities in subgroup analyses. Third, for the lacking of original data such as age, gender, family history and environment factors, our results were based on unadjusted estimates of ORs. Finally, since not all the investigations provided genotype counts separately by gastric cancer subtype, the pooled results derived from only a fraction of available studies may suffer from selection bias.

In conclusion, this meta-analysis indicated that the *PSCA* rs2294008 C>T and rs2976392 G>A polymorphisms might confer increased genetic susceptibility to cancer, especially bladder cancer and gastric cancer. However, due to the limitations of the meta-analysis, further prospective investigations with large sample size involving different ethnicities and gastric cancer subtypes are required to confirm these findings.

## MATERIALS AND METHODS

### Identification and eligibility of relevant studies

We systematically searched potential molecular epidemiology studies which investigated the association of *PSCA* gene polymorphisms with cancer risk through the electronic databases of Medline and Embase, using the following search terms: “*PSCA* or prostate stem cell antigen”, “polymorphism or variant or variation”, and “cancer or carcinoma or tumor or neoplasms'' (The last search was updated on April 18, 2015). We also searched additional publications written in Chinese from Chinese Biomedical (CBM) database to expand the coverage of our study. References cited in each of identified literatures were further searched manually for potential eligible studies. If studies had overlapped subjects, only the largest or latest one was adopted.

### Inclusion and exclusion criteria

Studies included had to meet the following criteria: (1) evaluating the association of *PSCA* rs2294008 C>T and/or rs2976392 G>A polymorphisms with cancer risk; (2) case-control or cohort designed; (3) sufficient genotype data provided for estimating odds ratios (ORs) and their corresponding 95% confidence interval (CIs); (4) written in English or in Chinese; (5) the control group of the studies should be in accordance with Hardy Weinberg Equilibrium (HWE).

The excluding criteria were as follows: (1) not relevant or not human subjects; (2) reviews or conference abstracts; (3) case only or survival only studies; (4) not written in English or Chinese; (5) providing duplicate data or overlapping with others.

### Data extraction

The following data were extracted from each eligible investigation: surname of first author, year of publication, country of origin, ethnicity, source of controls, cancer type, and subtype (intestinal or diffuse type for gastric cancer), cancer site [gastric cardia adenocarcinoma (GCA) or non-gastric cardia adenocarcinoma (NGCA) for gastric cancer], genotyping methods, total numbers of cases and controls, and the genotype counts for the *PSCA* rs2294008 C>T (CC/CT/TT) and the *PSCA* rs2976392 G>A (GG/AG/AA) polymorphism. Data were extracted independently by two investigators (Y.G. and R.-X. H.). A third investigator (P.-H. L.) would join to adjudicate any disagreement as needed. Ultimately, consensuses were reached on all the extracted information.

### Statistical methods

Based on the genotype frequency distribution in cases and controls, the associations of *PSCA* rs2294008 C>T and rs2976392 G>A polymorphism with cancer risk were measured by crude ORs and their corresponding 95% CIs for each study. The crude ORs and 95% CIs under the homozygous (TT vs. CC), heterozygous (CT vs. CC), recessive (TT vs. CT/CC), and dominant models (CT/TT vs. CC), as well as allele comparison model (T vs. C) were calculated for the rs2294008 C>T polymorphism, while homozygous model (AA vs. GG), heterozygous model (AG vs. GG), recessive model (AA vs. AG/GG), dominant model (AG/AA vs. GG), and allele comparison model (A vs. G) for the rs2976392 G>A polymorphism.

Goodness-of-fit chi-square test was conducted to check deviation from HWE among controls, and the deviation was significant at the 0.05 level. Between-study heterogeneity was assessed using chi-square-based Q statistical test. Generally, the fixed-effects model (Mantel–Haenszel method) [61] was adopted in the absence of heterogeneity. If *P* < 0.10, the random-effects model (DerSimonian–Laird method) [62], was chosen to calculate the pooled OR, since it takes the heterogeneity into account and yields wider CIs. Subgroup analyses were conducted by cancer type (less than three studies were categorized as others), ethnicity (Caucasians, Asians and Africans), source of control (hospital-based and population-based), subtypes (intestinal and diffuse) and sites (GCA and NGCA) if relevant data were available. An evaluation of publication bias was carried out using Egger's regression asymmetry test, Begg's rank correlation test and by visual inspection of the funnel plot [63]. In sensitivity analysis, each study was excluded at a time and the pooled ORs and 95% CIs were recalculated to determine the effect of each study on the summary risk estimate.

The statistical analysis was performed by using STATA version 11.0 (Stata Corporation, College Station, TX). All *P* values were two-sided, and *P* < 0.05 was considered as statistically significant.
